# Adaptive Shape Kernel-Based Mean Shift Tracker in Robot Vision System

**DOI:** 10.1155/2016/6040232

**Published:** 2016-06-09

**Authors:** Chunmei Liu, Yirui Wang, Shangce Gao

**Affiliations:** ^1^Department of Computer Science and Technology, Tongji University, Shanghai 201804, China; ^2^The College of Information Sciences and Technology, Donghua University, Shanghai 201620, China; ^3^Faculty of Engineering, University of Toyama, Toyama 930-8555, Japan

## Abstract

This paper proposes an adaptive shape kernel-based mean shift tracker using a single static camera for the robot vision system. The question that we address in this paper is how to construct such a kernel shape that is adaptive to the object shape. We perform nonlinear manifold learning technique to obtain the low-dimensional shape space which is trained by training data with the same view as the tracking video. The proposed kernel searches the shape in the low-dimensional shape space obtained by nonlinear manifold learning technique and constructs the adaptive kernel shape in the high-dimensional shape space. It can improve mean shift tracker performance to track object position and object contour and avoid the background clutter. In the experimental part, we take the walking human as example to validate that our method is accurate and robust to track human position and describe human contour.

## 1. Introduction

Object tracking is one major component in many robot domains as it directly affects the whole processing. Although significant amount of work has been done on object tracking in the literature, some difficulties still exist in objects tracking, such as nonrigid object structures, object occlusion, multiple connected objects, low contrast to the background, object scale variation, and complex object motion.

A large number of approaches have focused on object tracking. These works can be divided into three groups: point tracking, silhouette tracking, and kernel tracking. In [1], a point-based tracking approach is proposed by corresponding detected object points across frames. For tracking small objects, it is effective as it can use a single point to represent small objects, but for large objects it must use multiple points to represent large objects, which unavoidably leads to misdetection and occlusion. In comparison with the point-based tracking, a silhouette-based method focuses on an object shape description for tracking [2], which can flexibly handle a variety of object shapes. The kernel-based tracking approaches utilize a model region to represent the object in order to estimate object motion.

The kernel-based tracking approaches have many kinds of trackers. An early kernel-based visual tracker is the CAMSHIFT [3], which tracks the human faces by assigning each pixel a positive weight to find the location of a rectangular window in which the total weight of the pixels in the window is maximal. A kernel-based tracker [4] is proposed by minimizing a Bhattacharyya coefficient-based distance between the reference color distribution and the target's color distribution. An extended Kalman filter is suggested by updating the reference color histogram in [5]. The tracker in [6] was used by a mixture-of-Gaussians color model of the target. The affine object tracker is also performed in [7]. A kind of affine kernel-based trackers is presented by combining color-related kernel and boundary-related kernel to improve the tracking accuracy [8]. A kernel-based tracker is proposed by calculating the Gaussian pyramids of the images and applying mean shift algorithm at each pyramid level for the target [9]. Considering the computational complexity, it is proposed to use segmentation technique instead of using mean shift tracking algorithm [10]. Bolme et al. [11] exploit correlation filters based trackers to track visual objects.

Mean shift algorithm is an efficient kernel-based tracking approach which is a kind of nonparametric methods for seeking the nearest mode of a point sample distribution based on kernel density estimation [4, 12]. It is popular and widely applied in object tracking as it has many merits of low computation, easy implementation, real time response, and robust tracking performance.

It is challenging to have an ideal kernel with the shape adaptive to the object where no background points reside, especially the arbitrary shape of the nonrigid object. For the nonrigid object, it is very difficult to make the kernel shape the same as the object shape as the nonrigid object shape always varies. Some kernel-based methods are proposed to adapt to arbitrarily shaped object and overcome the background disturbance [13–17]. An asymmetric kernel mean shift algorithm is proposed to estimate object location, orientation, and scale by Yilmaz [14]. It introduces an implicit level set of functions to reduce the estimation bias and improve the density estimation process. The detected object mask is presented to construct a kernel by Yi et al. [16]. It is robust to background clutter and tracks the object very accurately if the object is detected accurately. So it depends heavily on the detection results. A GMM-SAMT algorithm is applied to achieve an asymmetric shape adapted kernel by Quast and Kaup [13]. An asymmetric kernel-based visual tracker is proposed by Leichter et al. [17]. It uses the target's color PDF to enhance the tracker's robustness.

**Figure 1:**
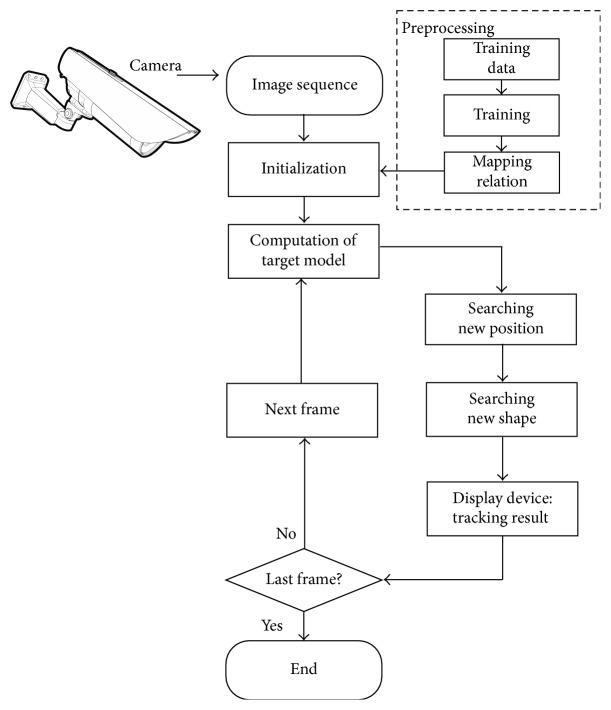
The flow chart of the proposed approach.

The work in this paper presents an adaptive shape kernel-based mean shift tracker to improve the position estimation and track the object contour under the environment captured by a static camera. The first contribution is the proposal of the adaptive shape kernel whose shape is reconstructed from the low-dimensional shape space to the high-dimensional shape space.  Figure 1 shows the whole algorithm architecture. In the preprocessing stage, we perform nonlinear manifold learning technique to obtain the mapping relation from the high-dimensional shape space to the low-dimensional shape space, which is trained by training dataset with the same view as the tracking video sequence. The second contribution of our paper is to work on a combination of the adaptive shape with color feature to describe the object's appearance. In contrast with the symmetric constant kernel used in the traditional tracker, it can better adapt to the object shape change to reduce the estimation error and improve the density estimation process. The whole processing is performed to find the right shape in the shape space and find the right position in position space for each frame in the video. Experiments demonstrate that this kind of tracker can outperform the traditional tracker significantly. Our method is accurate and robust to track object position and describe object contour especially when target shape deformation and background clutter occur.

The reminder of this paper is organized as follows. The traditional mean shift algorithm is reviewed in Section 2.  Section 3 explains how to construct an adaptive shape kernel from the embedding low-dimensional shape space to the high-dimensional shape space.  Section 4 introduces the process of the adaptive kernel shaped mean shift algorithm. In Section 5, the experimental studies are presented to prove the advantages of the proposed algorithm. Finally, Section 6 summarizes the main contributions of the paper together with discussions on some opening issues.

## 2. Mean Shift Tracking

Mean shift is a robust statistical algorithm, which applies a kernel density function in the new image based on the color histogram of the object in the previous image, and uses mean shift to find the maxima of a kernel density near the object's old position iteratively. It works with a search window that is positioned over a kernel density distribution. Within this search window, we compute the mean shift vector Δ*x* to evaluate the displacement of the object centroid. So the local maxima of the kernel density can be obtained by moving the searching window around the original position.

Firstly, we move the initial object position ${\overset{⌢}{x}}_{old}$ to a new position ${\overset{⌢}{x}}_{new}$ and repeat the average computation iteratively until the local maximum can be found. And the new position is updated: ${\overset{⌢}{x}}_{new}={\overset{⌢}{x}}_{old}+\Delta x$. The mean shift vector is computed as follows:

(1) Δx=∑Kx−x⌢oldwxx−x⌢old∑Kx−x⌢oldwx,wx=htIxhcIx,

where *h*_*t*_ and *h*_*c*_ are the color distribution functions generated from the target model and the candidate object region, respectively.

## 3. Adaptive Kernel Shape

Kernel shape is an important parameter of the mean shift algorithm, which decides which points participate in the computation. As all points in the kernel contribute to finding the local extrema, kernel shape plays an important part in mean shifting. The traditional mean shift method applies a symmetric kernel, such as a circle or an ellipse. This kind of kernel shape cannot match up with the object shape. The kernel window unavoidably covers many background points as well as the foreground object points. These background points inside the kernel window work as a part of the object. Therefore, the target tracking easily shifts to the wrong position as background clutter.

We consider making the kernel shape consistent with the object shape. However, it is not easy to describe the object shape accurately, especially for nonrigid object. During nonrigid object tracking, nonrigid object shape always varies. It becomes very crucial to keep the consistency between the kernel shape and the nonrigid object shape. In this paper, a kind of the adaptive kernel shape is proposed to describe the nonrigid object shape, which can avoid the disturbance of background points in the searching kernel window.

We use Γ to define the kernel shape parameter set. The transformed kernel parameters can be described as

(2) $$\begin{array}{c} P\left(\;x,y,\sigma ,\theta ,\Gamma \;\right), \end{array}$$

where (*x*, *y*) are the center coordinates of the tracking window position. *σ* is the scale dimension, *θ* is the orientation variation parameter, and Γ is the kernel shape parameter set. Here, we focus on the importance of the kernel shape parameters Γ. So the scale parameter *σ* and the orientation variation parameter *θ* are not considered in this paper.

In the following discussion, we provide the details on how to learn the adaptive kernel shape parameter set Γ. It is performed by three steps. Firstly, an object shape is represented by sampling points on the object contour. Secondly, we apply a nonlinear dimensionality reduction to transform the high-dimensional shape space into the low-dimensional shape space. This step includes two stages: training and tracking. In training stage, we use the training data which are required to have the similar view and the same object movement with the tracking object to obtain the low-dimensional shape space. Finally, in the tracking stage, the kernel shape of mean shift is reconstructed from the low-dimensional shape space to original shape space. In the following discussion, we take the walking human as example to illustrate the whole processing.

### 3.1. Shape Representation

It is necessary to represent precisely the object shape when tracking object. We can use some sampling points on the object contour to represent the object shape. The object has some key positions. These key position points are convex. These convex points play a significant role in the shape representation, which can be obtained by the projection of the object silhouette in some directions. These convex points can separate the shape contour into some parts. Each part has few singular points. It is flat. So we can sample uniformly the points on each part and use these sampling points to represent the object shape.

We take the walking human as example to illustrate how to handle the whole processing. For the walking human, human shape varies in a gait cycle in a camera's viewpoint. We represent each shape instance by sampling the points on the human contour. There are three key positions of human body: head point, left foot point, and right foot point to describe human shape. These three key positions are taken as reference points to separate human shape into three parts: head-right foot, right foot-left foot, and left foot-head.

**Figure 2:**
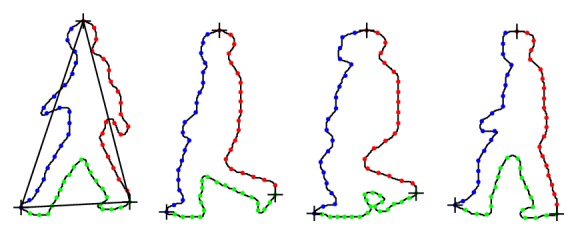
The shape points of silhouettes.

In order to find the head position, we projected the object silhouette onto the vertical axis (Figure 2). Head point is the top position of this projection. In the same way, the right part and the left part of the object silhouette are, respectively, projected onto the vertical axis. Left foot point and right foot point are, respectively, the bottom positions of projection.

After we obtain these three key points, we can separate the shape boundary into three parts: head-right foot, right foot-left foot, and left foot-head. For each part, 20 consecutive points are sampled. Thus, 60 points are employed to represent an instance shape.

### 3.2. Nonlinear Embedding

After sampling, the object shape can be represented in 60-dimensional space. However, it is difficult to search the right kernel shape in 60-dimensional shape space for the current instance. We provide a global geometric transformation for the shape instance between the low-dimensional shape space and the high-dimensional shape space. In this low-dimensional shape space, mean shift algorithm can search the right kernel shape window for the current instance.

As all points in the kernel contribute to finding the local extrema, kernel shape plays an important part in mean shifting. The traditional mean shift method applies a symmetric kernel, such as a circle or an ellipse. This kind of kernel shape cannot match up with the object shape. The kernel window unavoidably covers many background points as well as the foreground object points. These background points inside the kernel window work as a part of the object. Therefore, the target tracking easily shifts to the wrong position as background clutter.

For human walking, human shape has one cycle of the gait variation and suffers to deformation and self-occlusion which lead to the shape points lying on a nonlinear, twisted manifold. Isomap is a nonlinear dimensionality reduction method. It is one of the low-dimensional embedding methods, which provides a simple method for estimating the intrinsic geometry of a data manifold. It is highly efficient and generally applicable to a dimensionalities reduction. Here, we employ Isomap to embed 60 shape points in a 2-dimensional space.

**Figure 3:**
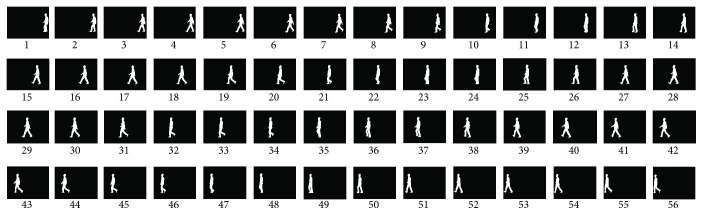
The silhouette sequence for training.

Given a set of *N*_*F*_ shape observations, *S* = {*s*_1_ ⋯ *s*_*N*_*F*__}, their corresponding embedding shapes are *T* = {*t*_1_ ⋯ *t*_*N*_*F*__}. Thus, each shape *s*_*i*_ can be represented as *t*_*i*_ in low-dimensional space.  Figure 3 shows an example of embedding walking cycles on a fixed view. We use a two-dimensional embedding space to describe the walking shape sequence in one cycle. As can be noticed, several embedding shape frames can represent a walking cycle.

### 3.3. Shape Reconstruction

When we obtain the low-dimensional shape space, it is possible to search the right kernel shape in the shape space. The searching of the kernel shape can be implemented in the low-dimensional shape space. However, if the right kernel shape was found, it is necessary to map the kernel shape from the low-dimensional space to the original high-dimensional space. Here, we can finish searching the kernel shape in the low-dimensional shape space. Similar to the mapping from the low-dimensional shape space into the original shape space, RBF is applied to reconstruct the shape in original shape space.

## 4. Adaptive Kernel-Based Mean Shift

As it is difficult to finish searching the right shape in high-dimensional shape space for tracking, kernel shape parameter is generally ignored. The points in the kernel participate in the computation to find the local extrema, so the kernel shape plays an important role in mean shift algorithm. An ideal kernel should have the same shape with tracking object without background clutter. However, as the dimensionality of shape space is very high, it is difficult to finish the shape searching in such high-dimensional space. Particularly, the shape of nonrigid object is a kind of arbitrary shape. It increases the difficulties of the kernel shape searching. In this paper, an adaptive shape based kernel is proposed for mean shift tracker. Firstly, the embedding low-dimensional shape space is found after training samples. In this embedding low-dimensional shape space, it is applied to finish searching the kernel shape for mean shift tracker. When the searching shape in low-dimensional space is identified, it is necessary to reconstruct it from low-dimensional space to original shape space. As mentioned above, RBF is applied to reconstruct the shape in original shape space.

We use Γ to define the kernel shape parameter set. Γ_*h*_  (*S* = {*s*_1_ ⋯ *s*_*N*_*F*__}) is the kernel shape parameter set in original shape space. In this paper, Γ_*l*_  (*T* = {*t*_1_ ⋯ *t*_*N*_*F*__}) is the shape parameter set in the embedding low-dimensional shape space. In the following discussion, we provide details on how to design adaptive kernel mean shift tracker, which combines adaptive kernel shape and color features to describe the object's appearance.

### 4.1. Color Feature

The proposed mean shift combines the reconstructed shape kernel and color features to find the most probable position of the target object through iteration. The detailed tracking process is implemented as follows.

The color histogram features can provide a better discriminating ability than gray histogram features. Here, *m* bin histograms in color space are selected to represent the objects' color probability density functions. So the color weighting histogram for the target model is obtained as follows:

(3) $$\begin{array}{c} {\overset{⌢}{q}}_{u}=C\overset{N}{\underset{i=1}{\sum }}K\left(\;•\;\right)\delta \left[\;b\left(\;x_{i}\;\right)-u\;\right], \end{array}$$

where *δ* is the delta function, *u* is the quantitative level of histograms, *u* = 1,…, *m*, *C* is the normalized constant, and *N* is the pixel number in the color-related kernel acting region. {*x*_*i*_^*∗*^}_*i*=1,…,*n*_ are the normalized pixel locations. *b*(*x*_*i*_^*∗*^) is the index of its bin in the quantized color feature space, *b*(*x*_*i*_) = 1,…, *m*. The function *K*(•) is the proposed kernel function which is based on the reconstructed kernel shape.

In the tracking window based on kernel shape, central pixels can provide more reliable information than boundary pixels for tracking as it suffers from less interference by background or occlusion. The distance from each pixel to the center of kernel shape is applied to compute its weight which decides the action rate for tracking matching.

Based on the reconstructed kernel shape, the adaptive kernel is computed by the normalization of each pixel distance *d*(*x*_*i*_) to the shape boundary:

(4) $$\begin{array}{c} K\left(\;x_{i}\;\right)=\frac{d\left(x_{i}\right)}{d_{max}}. \end{array}$$

Here, *d*_max_ is the maximum distance from the boundary to the center.

### 4.2. Implementation Process

The proposed mean shift combines the reconstructed shape kernel and color features to find the most probable position of the target object through iteration. The detailed tracking process is implemented as follows.

(1) Initialize the location of object *y*_0_ and the kernel shape Γ_0_ to compute color kernel *K*_*y*_0__ to obtain the target model *q*_*u*_.
(2) For a location of the tracking window *y*_*k*_, compute the model *p*_*k*_ by using previous color *K*_*y*_*k*−1__ based on shape Γ_*k*−1_ and estimate the new position ${\hat{y}}_{k}$ and update the weight *w*(*y*_*k*_):
  (5) y^k=∑i=1nhxiwigyk−xi/h2∑i=1nhwigyk−xi/h2,wi=∑u=1mqupuδbxi−μ.
(3) For a kernel shape of the tracking window *y*_*k*_, compute the model *p*_*k*_ by using previous color *K*_*y*_*k*__ based on shape Γ_*k*_ and estimate the new shape ${\hat{\Gamma }}_{k}$ by mapping between embedding shape space and original shape space.
(4) Track the object repeating steps (2)-(3).

## 5. Experiments

To demonstrate the performance of the proposed adaptive mean shift tracker, we have experimented with various sequences [18]. In all of the experiments with the proposed tracker, in the hsv color space the color-related component is used with equally spaced values in each color band, namely, with *m* = 2 × 2 × 16 bins, and the weight of the color feature kernel is set to 0.9.

### 5.1. Shape Reconstruction

**Figure 4:**
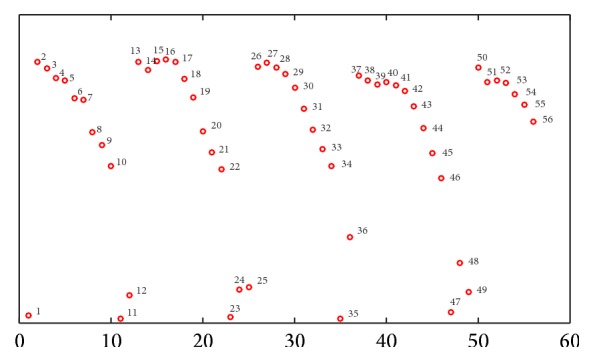
The embedded shape in 2-dimensional shape space.

**Figure 5:**
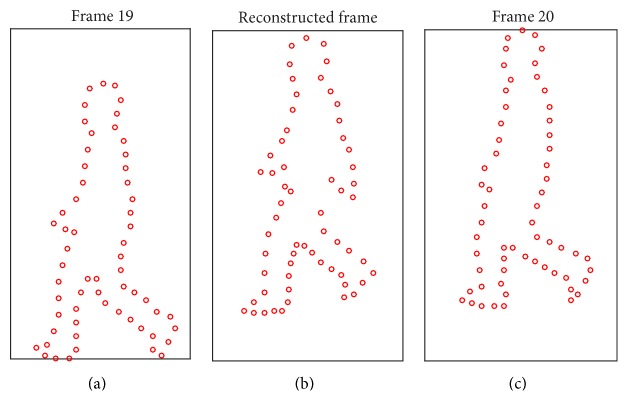
The reconstructed shape from 2-dimensional shape space to original shape space.

In all tested sequences, the initialization was manually performed in the first frame. The tracked shape is constructed from a training silhouette sequence in 0-degree direction (CASIA Gait Database [19]). As shown in Figure 3, the training silhouette sequence includes 56 frames, which corresponds to points in 2-dimensional shape space. Each point corresponds to one shape in original shape space. Furthermore, it demonstrates 12 shape points form one walking gait circle. The shape point on one walking circle corresponds to one shape in original shape, as shown in Figure 4. So it is executable to reconstruct any shape from 2-dimensional shape space to original shape.  Figure 5 shows the results. Figures 5(a) and 5(c) are original training shapes of frames 19 and 20.  Figure 5(b) is the reconstructed shape between training shapes of frames 19 and 20. So we can apply the proposed method to construct the shape of the arbitrary shape point in 2-dimensional shape space. It helps in searching shape parameter for kernel of mean shift tracker.

### 5.2. Bhattacharyya Coefficients Analysis

In order to demonstrate the robustness of the adaptive shape kernel-based mean shift to the background clutter, we implement a Bhattacharyya coefficients analysis comparing with the traditional mean shift algorithm based on a rectangular kernel shape. We make analysis of the Bhattacharyya coefficients which are used to find the similarity between the current tracking instance and the target model.

**Figure 6:**
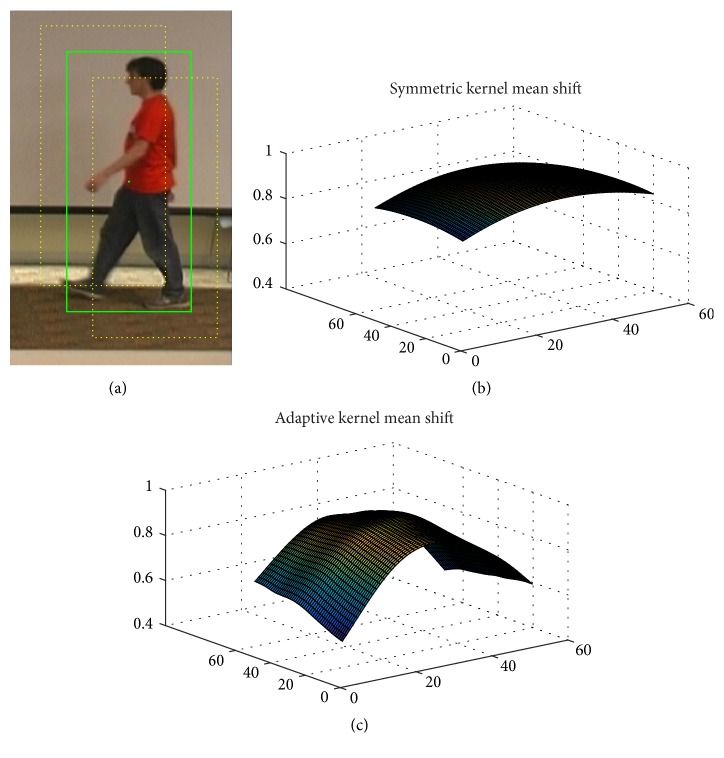
Bhattacharyya coefficients. (a) Tracking object; (b) Bhattacharyya coefficients result by symmetric kernel; and (c) Bhattacharyya coefficients result by adaptive kernel.

The Bhattacharyya coefficients are shown corresponding to the tracking windows centered in a 50 × 50 neighborhood around the object center [12] in Figure 6. In Figure 6(a), the green rectangle block is the object center area, and the yellow rectangle blocks are the tracking windows centered in the object center. Bhattacharyya coefficient 51 × 51 area is constructed by computing Bhattacharyya coefficients of the tracking windows as shown in Figures 6(b)-6(c). It is distributed convexly and monotonically. Generally the maximum corresponds to the object center.

Figure 6(b) shows that the Bhattacharyya coefficient surface by the ellipse shape is flat as there is less difference of Bhattacharyya coefficients between the object center and the object center's neighborhood. The reason is that the ellipse shape of kernel is inevitable to include some background points. These background points participate in the computation of Bhattacharyya coefficient. Thus this leads to the flat Bhattacharyya coefficient surface. In Figure 6(c), the Bhattacharyya coefficient surface by the proposed algorithm is steep because less background points participate in the computation of Bhattacharyya coefficient in the adaptive kernel of object center than the neighborhoods' of the object center. The kernel shape is adaptive to the object shape. There is less background influence in the adaptive kernel. So it is effective and robust when tracking the object.

### 5.3. Adaptive Shape Kernel-Based Mean Shift

The proposed algorithm works with the assumption that the video sequence to be processed is captured by a static camera. So in order to illustrate the performance of the proposed method, we tested three sequences from UIUC database [18] which have a similar view to training silhouette dataset. The video has been converted into sequences of image frames with resolution of 720 × 480 pixels.

**Figure 7:**
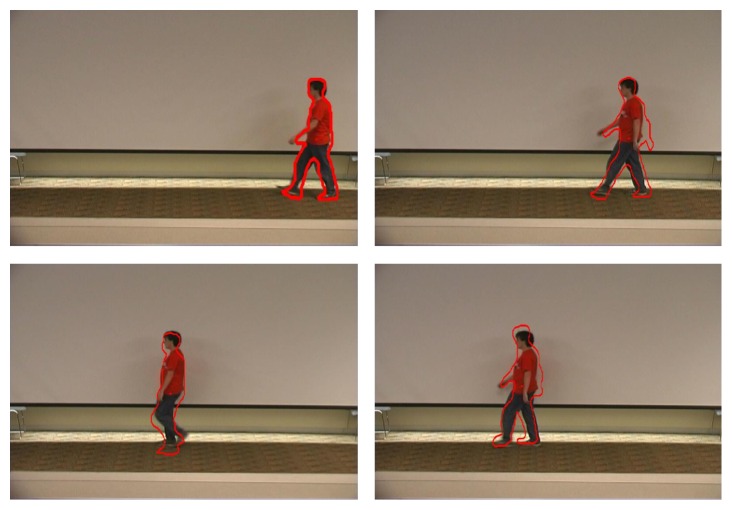
The results by adaptive kernel mean shift tracker.

We first present results for estimating the location and shape of a walking human.  Figure 7 shows four frames of one walking human sequence. As the proposed adaptive kernel can describe the object shape well and there is less background in the shape of an adaptive kernel, it is effective for human tracking. Compared with traditional mean shift tracker, it can not only track human position but also describe human contour. In Figure 7, the human contour tracking is not accurate enough as we performed position tracking and shape tracking only once for every frame in order to save computation time. If we performed the position searching once for each iterative shape during shape tracking, the results would be improved. But it would increase lots of computation. For the compromise considered, we apply the proposed iteration.

Here, we present the video sequences with the ground truth annotated by hand. We use precision and recall to evaluate the overall tracking performance for the proposed mean shift algorithm. In Table 1, our proposed method obtains overall tracking accuracy of 0.8~0.9. It demonstrates that the proposed algorithm can track not only the object position but also the object shape.

## 6. Conclusion

In this paper, we have proposed a novel adaptive kernel-based mean shift tracker, which integrates color feature kernel based on adaptive shape to improve object tracking performance. Experiments have validated that our method is accurate and robust to track human position and describe human contour. We believe that improvements are due to the increased accuracy of the kernel shape construction integrating color feature. We have noticed that the proposed method only adapts to the fixed camera view. In the future, we will try some other adaptive shape models for more views. On the other hand, recently considering performance and computation, the correlation filter-based trackers behave very well. A well-defined model shape helps in object tracking. We will further integrate the proposed adaptive shape with the correlation filter-based trackers to improve the tracking performance for robot vision system.

## Figures and Tables

**Table 1:** Overall tracking accuracy.

	Precision	Recall
*v*1	0.822	0.882
*v*2	0.818	0.8418
*v*3	0.801	0.8633

Precision = {true  shape}∩{tracked  shape}/{tracked  shape}.

Recall = {true  shape}∩{tracked  shape}/{true  shape}.
